# Beyond One-Trick Ponies: The Multifunctional Marvels of Microbial Functional Amyloids

**DOI:** 10.3390/microorganisms11051201

**Published:** 2023-05-04

**Authors:** Meytal Landau

**Affiliations:** 1 Department of Biology, Technion-Israel Institute of Technology, Haifa 3200003, Israel; mlandau@technion.ac.il; 2 Deutsches Elektronen-Synchrotron DESY, 22607 Hamburg, Germany; 3 Centre for Structural Systems Biology (CSSB), 22607 Hamburg, Germany; 4 Center for Experimental Medicine (ZEM), University Medical Center Hamburg-Eppendorf, 20246 Hamburg, Germany; 5 European Molecular Biology Laboratory (EMBL), 22607 Hamburg, Germany

Various organisms, including bacteria, protists, fungi, plants, and animals, secrete proteins and peptides that self-assemble into ordered amyloid fibrils that perform different physiological functions. In this Special Issue on Microbial Functional Amyloids, Balistreri et al. provide a comprehensive review of known functional amyloids and their wide range of functions, which likely represents only a foretaste of the actual number and activities of proteins that self-assemble into amyloids in all kingdoms of life [1]. The authors comprehensively describe the microbial amyloids that participate in virulent activities through highly orchestrated assemblies, focusing on *Escherichia coli* curli and *Pseudomonas aeruginosa* Faps, along with yeast prions. Álvarez-Mena et al. take us deeper into the multifunctionality of amyloids secreted by Gram-positive bacteria, including *Streptomyces coelicolor*, *Staphylococci*, *Streptococcus mutans*, *Bacillus* spp., and *Listeria monocytogenes* (Álvarez-Mena et al.) [2].

The function of amyloids as key virulence factors in microbes has rendered them attractive candidates for structural characterization aimed at the discovery of novel antimicrobial therapeutics. In contrast to the vast body of information available on eukaryotic amyloids involved in neurodegenerative and systemic diseases, mechanistic, functional, and high-resolution structural information on microbial amyloids is well-studied only for very specific systems. The research papers in this Special Issue reveal novel properties of virulent amyloid peptides from *S. aureus* (Zaman and Andreasen) [3] and of the major proteinaceous fibrillar biofilm component in *Bacillus subtilis* (Ghrayeb et al.) [4]. Both studies, which focused on very different amyloid systems, independently observed the modulation of fibrillation in response to changes in the environment. Zaman and Andreasen found a significant pH dependence of aggregation kinetics and fibril morphology of *S. aureus* phenol soluble modulins (PSMs). Such condition-specific behavior can modulate and switch between different roles, including cytotoxins, antimicrobial agents, and biofilm structuring. Ghrayeb et al. showed that native *Bacillus subtilis* TasA forms very different supramolecular morphologies when grown at neutral or acidic pHs, which was also dependent on variations in the concentration of the protein and salts [4]. The different fibril morphologies may encode different functional roles in biofilms. pH changes can also be used to switch between the storage and activity of toxic amyloids, as discussed for *L. monocytogenes* (Álvarez-Mena et al. [2]). The high-resolution structure of the TasA fibril was recently determined using cryogenic electron microscopy (cryo-EM), revealing fibrils that are distinct from typical amyloids but share a β-sheet-rich fiber morphology. Human amyloids are typically formed from molecules stacked perpendicular to the fibril axis to form paired β-sheets in a cross-β fibril. In contrast, TasA fiber is composed of folded monomers that are assembled by donor–strand exchange, with each subunit donating a β-strand to complete the fold of the next subunit along the fiber [5].

In addition to the multifunctionality of many amyloids, which may be encoded in different oligomeric and fibril morphologies controlled by environmental conditions, amyloid complexity is increased by interactions between different amyloids and with other components, such as proteins, lipids, nucleic acids, metal ions, and by post-translational modifications. Such interactions can be direct, between amyloids within the same species, as in the curli system (Balistreri et al.) [1], or cross-seeding between different microbes, as in the formation of dual-species biofilms (Balistreri et al. and Álvarez-Mena et al.) [1,2]. Interactions and cross-seeding between functional amyloids can be seen as an emerging theme in interspecies interactions contributing to the diversification of bacterial biology (Álvarez-Mena et al.) [2]. In addition, microbes and their amyloids may be involved in interactions with the human host (Balistreri et al. and Álvarez-Mena et al.) [1,2]. For example, evidence is accumulating for a still controversial link between human neurogenerative diseases and microbes and their amyloids. Balistreri et al. discussed the hypothesis that pathogens might induce amyloids associated with Parkinson’s disease. In general, microbes and their amyloids can induce various responses in the human host at the cellular, immunological, or physiological level (Balistreri et al. and Álvarez-Mena et al.) [1,2]. Microbial amyloid research would ultimately be used to combat aggressive infections and possibly processes leading to autoimmune and neurodegenerative diseases.

## Data Availability

No new data were created or analyzed in this study. Data sharing is not applicable to this article.
